# Foot-and-mouth disease virus-like particle vaccine incorporating dominant T and B cell epitopes: enhanced immune response in piglets with CD154 molecules

**DOI:** 10.3389/fvets.2025.1540102

**Published:** 2025-02-19

**Authors:** Yuwan Li, Weijun Zeng, Xinni Niu, Zhongmao Yuan, Shurou Li, Jinru Lin, Kaiyuan Xie, Zixiang Zhu, Lin Yi, Hongxing Ding, Mingqiu Zhao, Shuangqi Fan, Jinding Chen

**Affiliations:** ^1^College of Food and Bioengineering, Henan University of Science and Technology, Luoyang, Henan, China; ^2^Department of Preventive Veterinary Medicine, College of Veterinary Medicine, South China Agricultural University, Guangzhou, Guangdong, China; ^3^State Key Laboratory for Animal Disease Control and Prevention, College of Veterinary Medicine, Lanzhou University, Lanzhou Veterinary Research Institute, Chinese Academy of Agricultural Sciences, Lanzhou, China

**Keywords:** foot-and-mouth disease virus (FMDV), virus-like particle vaccine (VLP), CD154, T and B cell epitopes, ADDomer

## Abstract

**Introduction:**

Foot-and-mouth disease (FMD) is a highly contagious disease caused by FMDV, resulting in vesicular lesions in cloven-hoofed animals and posing significant economic threats to the livestock industry. VLP vaccines, which lack viral genetic material and are non-infectious, demonstrate superior safety compared to traditional inactivated vaccines. This study employs ADDomer, a novel adenovirus-based VLP framework, to display FMDV antigenic epitopes on the VLP surface. Additionally, FMDV capsid proteins can assemble into VLPs, offering innovative approaches for developing more efficient and safer FMDV vaccines.

**Methods:**

Two FMDV VLP proteins were constructed using a baculovirus expression system. One VLP was developed by embedding the B-cell epitope of FMDV VP1 into the G-H loop of VP3 and co-expressing it with VP1 and VP0 to form VP1-VP3_B_-VP0. The other VLP, ADDomer-BBT, fused B-and T-cell epitopes from FMDV O-type VP1 into the ADDomer platform, with porcine CD154 expressed as an immune enhancer. Expression conditions were optimized, and proteins were purified. The VLPs, combined with porcine CD15 molecular adjuvant, were evaluated for immunogenicity in piglets.

**Results:**

After purification, both VLPs displayed virus-like structures under electron microscopy. Immunization in piglets induced high levels of FMDV-specific and neutralizing antibodies, enhanced cytokines IL-2, IL-4, and IFN-γ, and increased lymphocyte proliferation. The CD154-added group showed higher immune responses.

**Discussion:**

The VLP vaccines effectively induced strong cellular and humoral immune responses, with CD154 enhancing efficacy. These findings provide insights for developing safer, more effective FMDV vaccines and contribute to advancing livestock health and productivity.

## 1 Introduction

Foot-and-mouth disease virus (FMDV), a single-stranded positive-sense RNA virus, belongs to the genus *Aphthovirus* within the *Picornaviridae* family. The virions display an icosahedral structure with a diameter of ~30 nm (1). FMDV is known for its remarkable infectivity, capable of entering the host through nasal, oral, and even airborne routes (2, 3). Due to its extensive global prevalence and high transmissibility, FMD outbreaks often cause devastating impacts on local livestock industries (4–6). Vaccination is considered an effective method for controlling FMD. However, traditional inactivated vaccines face challenges related to stability, safety, and production costs. Therefore, the development of novel, genetically engineered FMDV vaccines to prevent and eradicate FMD is urgently needed (7). Among FMDV structural proteins, VP1, VP2, VP3, and VP4 play critical roles in viral capsid assembly, particle stability, and antigen specificity (8, 9). Of particular interest, VP1 contains multiple essential antigenic epitopes, with some B-cell and T-cell epitopes from VP1 known to induce cellular and humoral immune responses when used in epitope peptide vaccines (10–12). However, these simple peptide vaccines often exhibit limited immunogenicity and require adjuvants to enhance the immune response.

Virus-like particles (VLPs) are hollow particles assembled from one or more viral proteins that lack viral nucleic acids. Due to the absence of genetic material, VLPs are incapable of replication, thereby offering a high degree of safety (13). Their structural similarity to native viruses enables them to elicit a strong immune response (14, 15). VLPs can originate from viruses that infect humans, animals, plants, and bacteria, and are primarily formed by the self-assembly of viral capsid proteins. For instance, the co-expression of FMDV capsid proteins VP0, VP1, and VP3 can spontaneously assemble into VLPs. Similarly, the Cap protein of porcine circovirus type 2 (PCV-2) and the VP2 protein of porcine parvovirus (PPV) can also self-assemble into VLPs in vitro. For viruses that cannot self-assemble into particles, “chimeric” technology can be employed to present antigens on specific particle carriers to prepare chimeric VLPs. For example, a recombinant T7 phage VLP vaccine incorporating the VP1 protein of FMDV through phage display technology can stimulate a robust immune response in the host (16). Recently, researchers developed a novel multi-epitope VLP display platform, the ADDomer, based on an adenovirus-derived nanoparticle framework with excellent thermal stability. The platform, characterized by unique VL and RGD regions, allows for high-density presentation of diverse chimeric antigens on the ADDomer surface, significantly enhancing the stability and immunogenicity of short peptide vaccines. Prior clinical trials have demonstrated that a VLP vaccine incorporating the Chikungunya virus E2EP3 epitope into the RGD region of ADDomer exhibited exceptional immunogenicity (17). Amidst the SARS-CoV-2 pandemic, researchers effectively leveraged the ADDomer platform to achieve high-density surface presentation of the SARS-CoV-2 receptor-binding domain (RBD), developing a SARS-CoV-2 VLP candidate vaccine that successfully elicited high levels of neutralizing antibodies in mice (18). Other studies have highlighted that adding adjuvants could further improve VLP vaccine immunogenicity (19–21). CD154, a specific ligand for CD40, plays a crucial role in modulating both humoral and cellular immune responses (22–24). Notably, research has shown that CD154 as a molecular adjuvant significantly enhances immune responses in both porcine circovirus and human respiratory syncytial virus vaccines (25, 26).

Based on these findings, this study utilized a baculovirus expression system to prepare two types of FMDV VLPs. One VLP was generated by embedding the FMDV B-cell epitope into the variable G-H loop region of VP3, co-expressed with VP1 and VP0 recombinant proteins. The other VLP was constructed by incorporating B- and T-cell epitopes from the FMDV O-type VP1 protein into the ADDomer display platform. Both VLPs were combined with the molecular adjuvant CD154 to evaluate immunogenic efficacy in piglets, aiming to provide new insights into FMDV vaccine development and adjuvant research.

## 2 Materials and methods

### 2.1 Cell culture and viral infections

SF9, High Five, and BHK-21 cells were maintained in the Veterinary Microbiology and Immunology Laboratory at South China Agricultural University. SF9 and High Five cells were cultured in SIM SF Expression Medium and SIM HF Expression Medium (Sino Biological Inc., Beijing, China), respectively, under suspension conditions in a 27°C incubator shaker at 140 rpm. BHK-21 cells were cultured in DMEM medium containing 10% fetal bovine serum (Gibco, USA) at 37°C with 5% CO_2_ in a cell incubator. FMDV type O strain O/BY/CHA/2010 was propagated in BHK-21 cells using standard virology techniques and the supernatants of infected cells were clarified and stored at −80°C.

### 2.2 Design and synthesis of recombinant protein gene sequences

**Figure 1:**
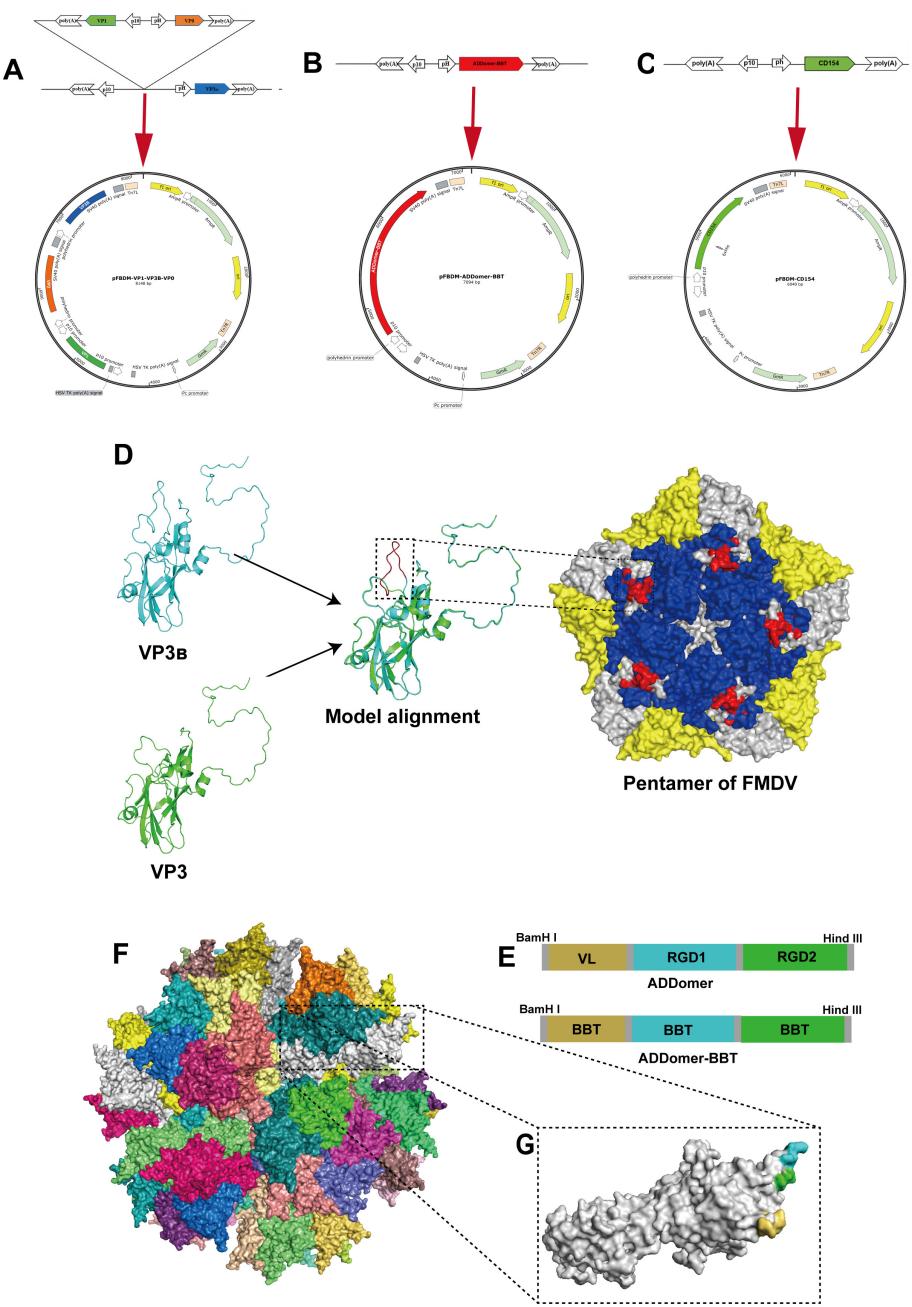
Design of recombinant proteins. **(A–C)** Illustrate schematic diagrams of the recombinant transfer vectors pFBDM-VP1-VP3_B_-VP0, pFBDM-ADDomer-BBT, and pFBDM-CD154, respectively. **(D)** Shows the structure of VP3_B_ with the inserted B-cell epitope and homology modeling of the FMDV capsid protein pentamer (VP1, VP2, and VP3_B_ regions in blue, yellow, and white, respectively), with the inserted epitope highlighted in red within the black box. **(E)** Shows the insertion site design within the ADDomer framework. **(F)** Depicts the simulated assembled ADDomer virus-like particle (VLP) composed of 60 monomers. **(G)** Shows an ADDomer monomer with VL (yellow), RGD1 (blue), and RGD2 (green) regions.

Based on the VP1, VP3, and VP0 gene sequences of the FMDV type O reference strain (GenBank: JN998085.1), a histidine (His) tag sequence encoding six histidines was introduced at the C-terminus of each protein. A dominant B-cell epitope (amino acids 200–213) was inserted into the G-H loop (positions 173–174 aa) of the VP3 protein, resulting in the gene sequences for VP1, VP3B, and VP0. Additionally, two B-cell epitopes (137–160aa and 200–213aa) and one T-cell epitope (21–40aa) from VP1 were sequentially linked together and then inserted into the VL, RGD1, and RGD2 regions of the ADDomer (Patent No. US201716088905). A His tag was added at the end of the T-cell epitope to generate the ADDomer-BBT protein gene sequence. The predicted structure of the recombinant VLP was modeled using the crystal structure files with PDB IDs 8HEE (FMDV) and 6HCR (ADDomer) and the online homology modeling website Swiss Model (http://swissmodel.expasy.org/; accessed on October 10, 2024), with structural visualization performed using PyMOL software.Based on the porcine CD154 gene sequence (GeneBank: NM_214126.1), a His tag sequence encoding six histidines was introduced at the C-terminus, resulting in the recombinant protein gene sequence for CD154. To ensure high expression levels in insect cells, the codons of the five gene sequences were optimized and synthesized by Hongxun Biotechnology Co., Ltd. (Suzhou, China). The sequences were inserted into the pFBDM vector as shown in Figures 1A–C.

### 2.3 Generation of recombinant baculovirus

The constructed recombinant transfer plasmids pFBDM-VP1-VP3_B_-VP0, pFBDM-ADDomer-BBT, and pFBDM-CD154 were transformed into DH10MultiBac competent cells. The transformed cells were spread on LB agar plates containing Gen, Kan, Amp, IPTG, and X-Gal and incubated at 37°C for 48 h. White colonies were selected, and PCR identification was performed using the M13 universal primers of the pFBDM vector. The positively identified recombinant colonies were expanded and used for recombinant baculoviral plasmid extraction. The extracted recombinant baculoviral plasmids were designated as rFBDM-VP1-VP3_B_-VP0, rFBDM-ADDomer-BBT, and rFBDM-CD154. Following Wu et al.'s method (26), the recombinant baculovirus plasmids were transfected into SF9 cells. After 96 h incubation at 27°C, cytopathic effects were observed, and the supernatants were collected as P1 recombinant baculoviruses, named Ac-VP1-VP3_B_-VP0, Ac-ADDomer-BBT, and Ac-CD154, respectively.

### 2.4 Titration of recombinant baculovirus

To quantify the recombinant baculovirus concentration, absolute fluorescence quantitative PCR was performed. Standard pMD18T-Ac plasmids at concentrations of 1 × 10^3^ to 1 × 10^8^ copies/μl were used to create a standard curve. Viral DNA was extracted from Ac-VP1-VP3_B_-VP0, Ac-ADDomer-BBT, and Ac-CD154 recombinant baculoviruses according to the Viral DNA Kit instructions, serving as the template for fluorescence quantitative PCR. The virus copy number was calculated using the standard curve, and the viral titer was determined using Equation 1.


(1) $$\text{Viral titer }(\text{pfu/ml})\text{ = }\frac{\text{DNA copy number }(\text{copies/μl})\;\times {\text{10}}^{\text{3}}}{\text{53}}$$

### 2.5 Expression and identification of recombinant proteins

The obtained recombinant baculoviruses were passaged to P3 and analyzed by Western blot. Primary antibodies included mouse monoclonal anti-His (Sino Biological Inc., Beijing, China), porcine FMDV-positive serum (Yongshun Biotechnology Co., Ltd., Guangdong, China), rabbit monoclonal anti-FMDV VP1 (Bioss Biotechnology Co., Ltd., Beijing, China), and recombinant anti-TARP/CD40L (Abcam plc, UK). Secondary antibodies were goat anti-mouse and goat anti-rabbit IgG-HRP antibodies (Beyotime Biotechnology Co., Ltd., Shanghai, China). PVDF membranes were visualized using ECL chemiluminescent solution (YaMei Biological Medicine Co., Ltd., Shanghai, China) with a gel imaging system.

Due to the lack of VP3 and VP0 monoclonal antibodies, mass spectrometry was performed on all three proteins. P3-generation SF9 cells infected with Ac-VP1-VP3_B_-VP0 for 72 h were harvested, mixed with protein sample buffer, and denatured in a metal bath at 100°C for 10 min before SDS-PAGE electrophoresis. The gel was stained with BeyoBlue™ Coomassie Brilliant Blue, and the protein bands of expected sizes were excised, placed in 1.5 ml EP tubes, and sent to Biopeson Biotechnology Co., Ltd. (Shanghai, China) for protein identification via mass spectrometry.

To further verify recombinant protein expression, SF9 cells were infected with P3-generation Ac-VP1-VP3_B_-VP0, Ac-ADDomer-BBT, and Ac-CD154 at an MOI of 0.1, with a negative control group (uninfected). After 72 h at 27°C, indirect immunofluorescence detection was performed using a 1:250 diluted mouse anti-His antibody as the primary antibody and a 1:1,000 diluted FITC-labeled goat anti-mouse IgG antibody as the secondary antibody.

### 2.6 Optimization of recombinant protein expression conditions

To determine optimal harvest time and MOI for recombinant protein expression, High Five cells were used for large-scale expression, and P3 recombinant baculoviruses were inoculated at MOI values of 2.0, 5.0, and 8.0. After shaking at 27°C and 130 rpm, samples were collected at 48 h, 72 h, 96 h, and 120 h post-infection and analyzed by Western blot. Protein gray values were quantified with ImageJ software, and expression trend graphs were generated to determine the optimal MOI and harvest time in High Five cells.

### 2.7 Sucrose gradient centrifugation purification of VLPs

Based on the optimized expression conditions in 2.6, Ac-VP1-VP3_B_-VP0, Ac-ADDomer-BBT, and Ac-CD154 P3 recombinant baculoviruses were inoculated into High Five cells for large-scale protein expression. After ultrasonic disruption of collected cells, supernatants were centrifuged at 12,000 rpm for 20 min at 4°C and filtered using Millipore ultrafiltration tubes. Samples were loaded onto sucrose gradients of 70%, 50%, and 30% and centrifuged at high speed. The resulting protein bands were collected, filtered through 0.22 μm membranes, and analyzed by SDS–PAGE. The gels were stained with BeyoBlue™ Coomassie Brilliant Blue to evaluate sucrose gradient centrifugation purification results.

To investigate the morphology of the recombinant proteins, 10 μl samples were added to carbon-coated grids, stained with 2% uranyl acetate, and dried for observation under a transmission electron microscope (TEM).

### 2.8 Semi-quantitative analysis of recombinant proteins

A His-tagged protein at 2,000 μg/ml was serially diluted and used as a standard alongside the recombinant protein samples for Western blot analysis. ImageJ was used to analyze the gray values of the standards, and a standard curve was generated to calculate recombinant protein concentrations.

### 2.9 Preparation and immunization of FMDV VLP vaccines

The concentrations of recombinant proteins VP1-VP3_B_-VP0, ADDomer-BBT, and CD154 were adjusted with PBS buffer. The proteins were mixed with ISA 201 (SEPPIC, France) adjuvant at a 1:1 mass ratio to produce vaccines with final dosages as follows: 70 μg VP1, 50 μg VP3, and 30 μg VP0 per 2 ml dose for the FMDV VLP vaccine (VP1-VP3_B_-VP0 vaccine); 50 μg of ADDomer-BBT recombinant protein per 2 ml dose for the FMDV chimeric VLP vaccine (ADDomer-BBT vaccine); and 25 μg of recombinant CD154 as a molecular adjuvant in the molecular adjuvant control groups (VP1-VP3_B_-VP0 + CD154 and ADDomer-BBT + CD154). A control was prepared with PBS and ISA 201 adjuvant in a 1:1 ratio. The vaccines were assessed for appearance, formulation, and stability.

Thirty 4-week-old seronegative piglets were randomly divided into six groups (five piglets per group) as shown in Table 1. After being raised for 1 week, a primary immunization was administered by intramuscular injection. The piglets were observed daily for local and systemic adverse reactions following vaccination.

**Table 1:** Piglet immunization plan design.

**Group**	**Type and composition**	**Immunization dose and method**
1. VP1-VP3_B_-VP0	W/O/W, ISA 201 VG	2 ml (70 μg+50 μg+30 μg)/pig; im
2. VP1-VP3_B_-VP0 + CD154	W/O/W, ISA 201 VG	2 ml (70 μg+50 μg+30 μg+ 25 μg)/pig; im
3. ADDomer-BBT	W/O/W, ISA 201 VG	2 ml (50 μg)/pig; im
4. ADDomer-BBT + CD154	W/O/W, ISA 201 VG	2 ml (50 μg + 25 μg) /pig; im
5. Vaccine (commercialized vaccine)	W/O/W, ISA 201 VG	2 ml/pig; im
6. PBS	PBS	2 ml/pig; im

### 2.10 Detection of FMDV-specific antibodies in piglet peripheral serum

FMDV-specific antibody levels in the peripheral blood of piglets were determined using the Porcine FMDV O-type Antibody ELISA Kit, following the manufacturer's instructions (Finder Biotechnology Co., Ltd., Shenzhen, China).

### 2.11 Neutralizing antibody titer assay in piglet peripheral serum

Healthy BHK-21 cells were seeded into 96-well plates and cultured in a 5% CO_2_ incubator at 37°C until 80% confluency. Serum samples collected on day 35 post-initial immunization were heated at 56°C for 30 min and serially diluted in DMEM containing 2% FBS. A mixture of 300 μl of the diluted serum and 300 μl of FMDV suspension containing 100 TCID50 was incubated at 37°C for 1 h. Subsequently, 100 μl of the virus-serum mixture was added to each well of the prepared BHK-21 cells, with a blank negative control included. Each dilution level was tested in quadruplicate, and the plates were incubated at 37°C in a 5% CO_2_ environment for 6–8 h. After incubation, the supernatant was removed, and serum-free DMEM was added to each well, with each group set up in four replicates. Cytopathic effects (CPE) were monitored under a microscope, and the neutralizing antibody titer was calculated based on the highest serum dilution exhibiting 50% CPE.

### 2.12 Piglet peripheral blood lymphocyte proliferation assay

Peripheral blood lymphocytes from piglets were isolated from blood samples collected on day 35 post-initial immunization using a lymphocyte separation kit (HaoYang Biological Products Technology Co., Ltd., Tianjin, China) according to the manufacturer's instructions. The lymphocyte concentration was adjusted to 5 × 10^6^ cells/ml. Lymphocytes were divided into positive control, negative control, and experimental groups, with each group set up in four replicates. Cells were seeded at 100 μl per well. The positive control group received 100 μl of Con A (10 μg/ml), the negative control received 100 μl of complete 1640 medium with 10% FBS, and the experimental group received 100 μl of inactivated FMDV. After 48 h of incubation at 37°C in a 5% CO_2_ incubator, 10 μl of CCK-8 solution was added to each well and incubated for an additional 2–4 h. The OD450 value was measured every 30 min, and the stimulation index (SI) was calculated as SI = average OD450 (virus-stimulated or positive control group)/average OD450 (1640 complete medium group).

### 2.13 Cytokine assay in piglet peripheral blood

The levels of IFN-γ, IL-2, and IL-4 in peripheral blood from piglets were measured using ELISA kits for porcine IFN-γ (RX501082P), IL-2 (RX501069P), and IL-4 (RX501065P) in accordance with the manufacturer's instructions (Ruisen Biotechnology Co., Ltd., Fujian, China).

### 2.14 Statistical analysis

Data were analyzed using GraphPad Prism 9 software, applying one-way ANOVA or two-way ANOVA for statistical comparisons between groups. A probability (*P*) value of < 0.05 was considered statistically significant.

## 3 Result

### 3.1 Design and construction of recombinant vectors

The structure of the VP3_B_ protein with a B-cell epitope inserted in the G-H loop was predicted and compared to the unmodified VP3 structure. The results showed that the inserted epitope in the central region of the G-H loop was exposed on the protein's surface (Figure 1D). Moreover, tandem antigenic epitopes were inserted into the ADDomer framework based on the insertion pattern in Figure 1E. The inserted epitopes in the VL, RGD1, and RGD2 regions are all displayed on the particle's outer surface (Figures 1F, G). The designed sequences were synthesized and inserted into the pFBDM vector (Figures 1A–C).

### 3.2 Generation of recombinant baculovirus and viral titer determination

**Figure 2:**
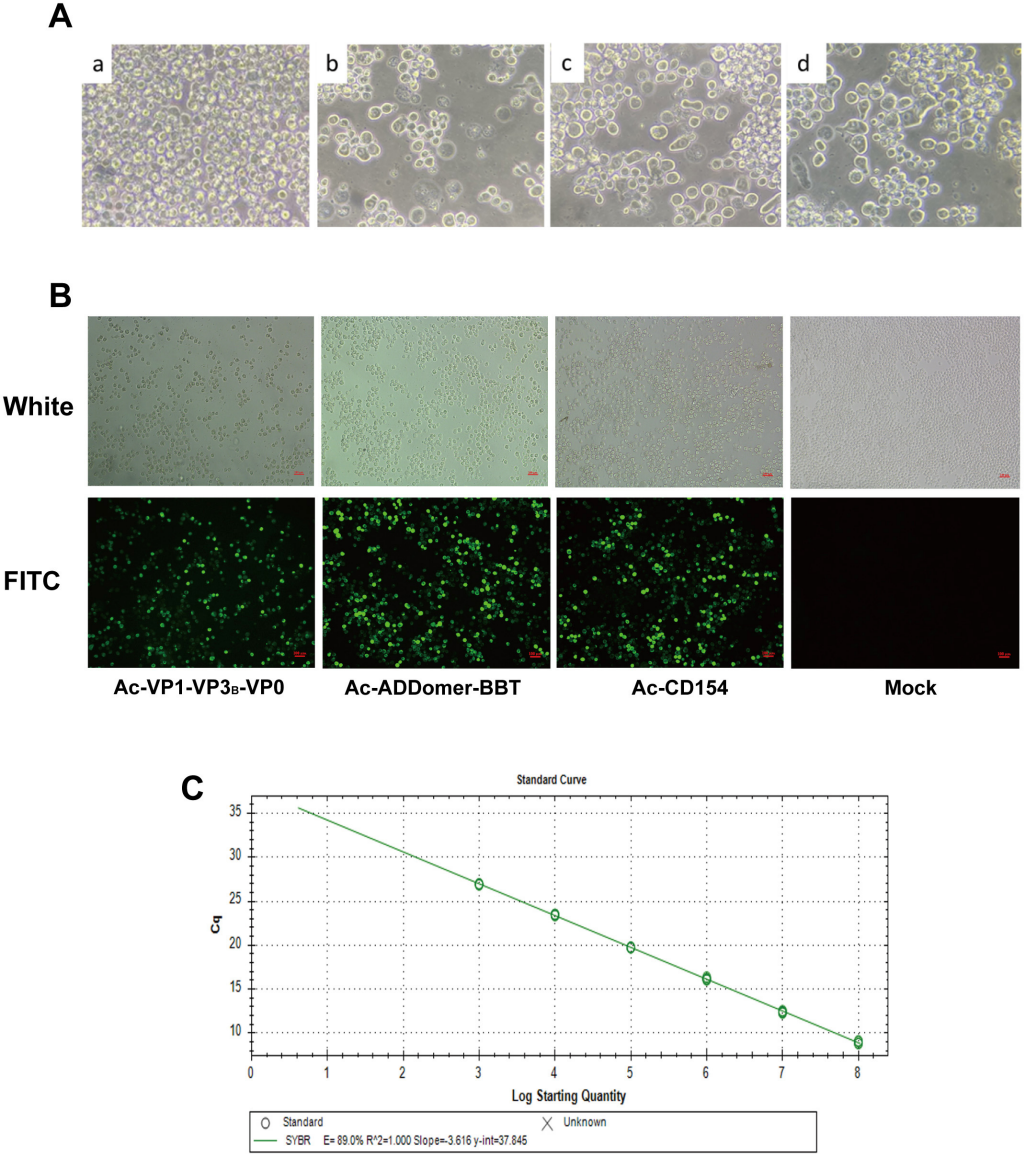
Construction of Recombinant Baculovirus Particles. **(A)** SF9 cells post-transfection vs. normal cells: a represents normal SF9 cells, while b, c, and d represent SF9 cells transfected with recombinant baculovirus plasmids rFBDM-VP1-VP3B-VP0, rFBDM-ADDomer-BBT, and rFBD-CD154, respectively. **(B)** Indirect immunofluorescence assay of recombinant protein expression. **(C)** Standard curve for baculovirus plasmid copy number quantification.

Recombinant baculoviral plasmids rFBDM-VP1-VP3_B_-VP0, FBDM-ADDomer-BBT, and rFBDM-CD154 were transfected into adherent SF9 cells in the logarithmic growth phase. After incubation at 27°C for 72 h, cell morphology was observed. As shown in Figure 2A, compared to untransfected cells, SF9 cells transfected with recombinant baculovirus plasmids exhibited extensive detachment and cell death, with a marked increase in cell volume, nuclear swelling, and blurred cell membrane edges. The cell culture supernatant was collected, centrifuged, and used to obtain the P1 generation recombinant baculovirus, designated as Ac-VP1-VP3_B_-VP0, Ac-ADDomer-BBT, and Ac-CD154.

IFA analysis of SF9 cells infected with the P3 generation of recombinant baculovirus revealed specific green fluorescence in cells infected with Ac-VP1-VP3_B_-VP0, Ac-ADDomer-BBT, and Ac-CD154, while no fluorescence was detected in the negative control group, confirming the successful construction of recombinant baculovirus capable of expressing recombinant proteins (Figure 2B). Absolute quantitative PCR with established standards produced linear standard curves (Figure 2C), and the Cq values were used to calculate recombinant baculovirus copy numbers for P3 Ac-VP1-VP3_B_-VP0, Ac-ADDomer-BBT, and Ac-CD154, yielding 2.55 × 10^8^, 3.972 × 10^8^, and 1.191 × 10^8^ copies/μl, respectively. Then, the viral titers were calculated according to the formula, resulting in titers of 4.811 × 10^9^ pfu/ml, 7.494 × 10^9^pfu/ml, and 1.767 × 10^9^ pfu/ml.

### 3.3 Characterization of recombinant protein expression

**Figure 3:**
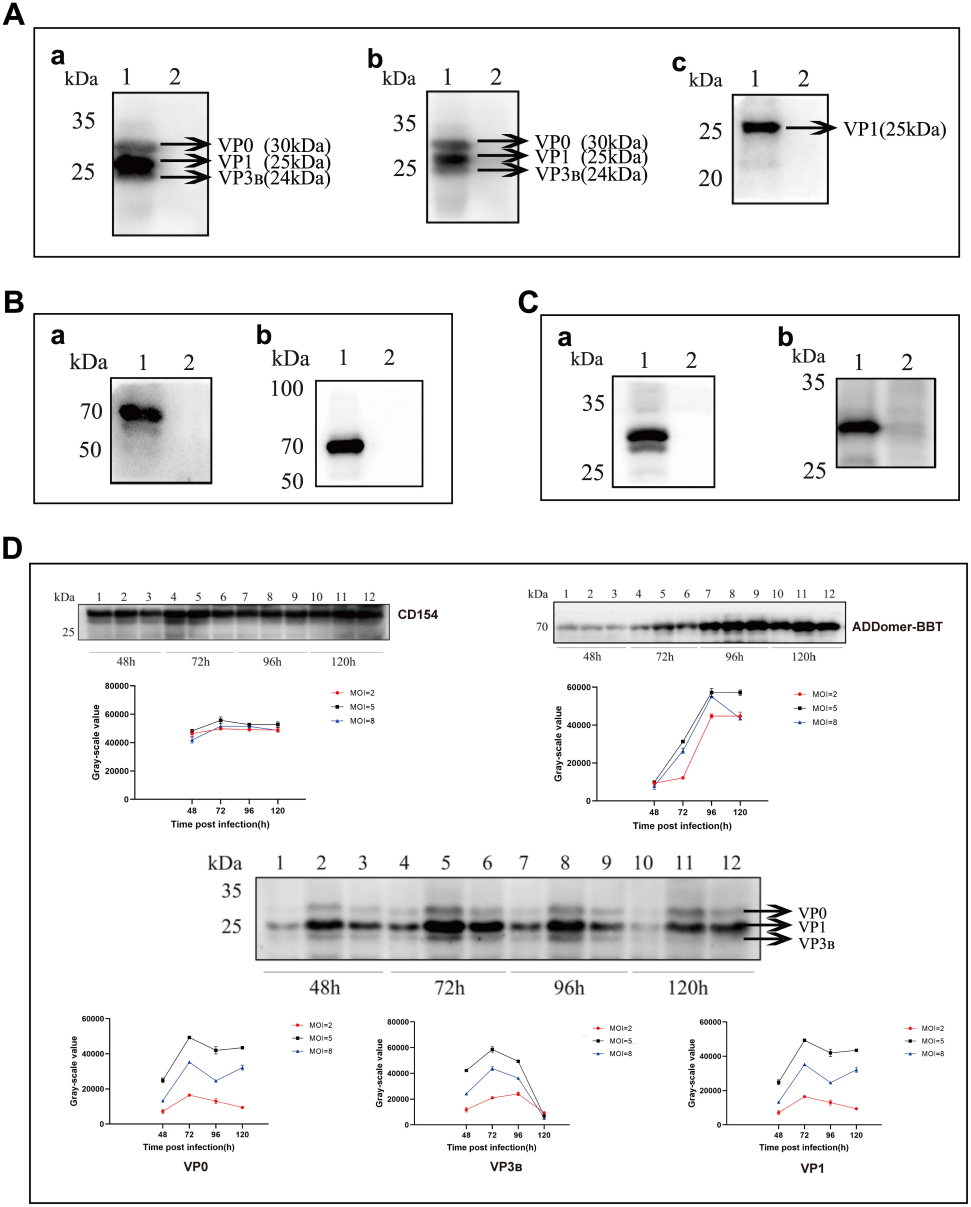
Expression and Characterization of Recombinant Proteins. **(A)** Western blot analysis of the FMDV VP1-VP3_B_-VP0 protein. (a) Using mouse anti-His tag antibody as the primary antibody. (b) Using FMDV-positive serum as the primary antibody. (c) Using rabbit anti-FMDV VP1 monoclonal antibody as the primary antibody. Lane 1: Lysate of SF9 cells infected with Ac-FMDV-VP1-VP3_B_-VP0. Lane 2: Negative control. **(B)** Western blot analysis of the ADDomer-BBT protein. (a) Using mouse anti-His tag antibody as the primary antibody. (b) Using rabbit anti-FMDV VP1 monoclonal antibody as the primary antibody. Lane 1: Lysate of SF9 cells infected with Ac-ADDomer-BBT. Lane 2: Negative control. **(C)** Western blot analysis of the CD154 protein. (a) Using mouse anti-His tag antibody as the primary antibody. (b) Using recombinant anti-TARP/CD40L antibody as the primary antibody. Lane 1: Lysate of SF9 cells infected with Ac-CD154. Lane 2: Negative control. **(D)** Optimization of recombinant protein expression conditions.

Using a mouse-derived anti-His tag antibody and FMDV-positive serum as primary antibodies, the recombinant VP1-VP3_B_-VP0 protein expression was identified by Western blot analysis, with specific bands observed at ~24 kDa, 25 kDa, and 30 kDa (Figure 3A). To further confirm VP1-VP3_B_-VP0 protein expression, mass spectrometry analysis was performed using the original files processed with MasQuant 2.0.1.0 based on the supplementary parameters in Table 2, verifying the presence of VP1, VP3_B_, and VP0. The mass spectrometry results are shown in Supplementary Figure S1. For the ADDomer-BBT recombinant protein, Western blot analysis using mouse-derived anti-His and rabbit-derived anti-FMDV VP1 monoclonal antibodies as primary antibodies showed a specific band at 71 kDa (Figure 3B). For the recombinant CD154 protein, Western blot analysis using mouse-derived anti-His and anti-TARP/CD40L antibodies as primary antibodies revealed a specific band at 30 kDa (Figure 3C).

**Table 2:** Database retrieval parameters.

**Enzyme**	**Trypsin**
Max missed cleavages	2
Precursor tolerance (main search)	4.5 ppm
Precursor tolerance (first search)	20 ppm
MS/MS tolerance	20 ppm
Fixed modifications	Carbamidomethyl (C)
Variable modifications	Oxidation (M), acetyl (protein N-term)
Database	Uniprot-Sus scrofa (pig)
Database pattern	Target-reverse
PSM FDR	0.01
Protein FDR	0.01
Site FDR	0.01

P3 generation recombinant baculovirus was inoculated into suspension High Five cells to optimize the inoculum amount and protein harvest time. Figure 3D shows the results. For practical production cost considerations, an MOI of 5.0 was selected as the optimal dose for Ac-CD154 recombinant baculovirus, with CD154 protein harvested at 72 h post-inoculation. For Ac-ADDomer-BBT, the optimal MOI was 5.0, with ADDomer-BBT protein harvested at 96 h post-inoculation. Similarly, an MOI of 5.0 was used for Ac-VP1-VP3_B_-VP0, with protein harvested at 72 h.

### 3.4 TEM observation and semi-quantitative analysis of recombinant proteins

**Figure 4:**
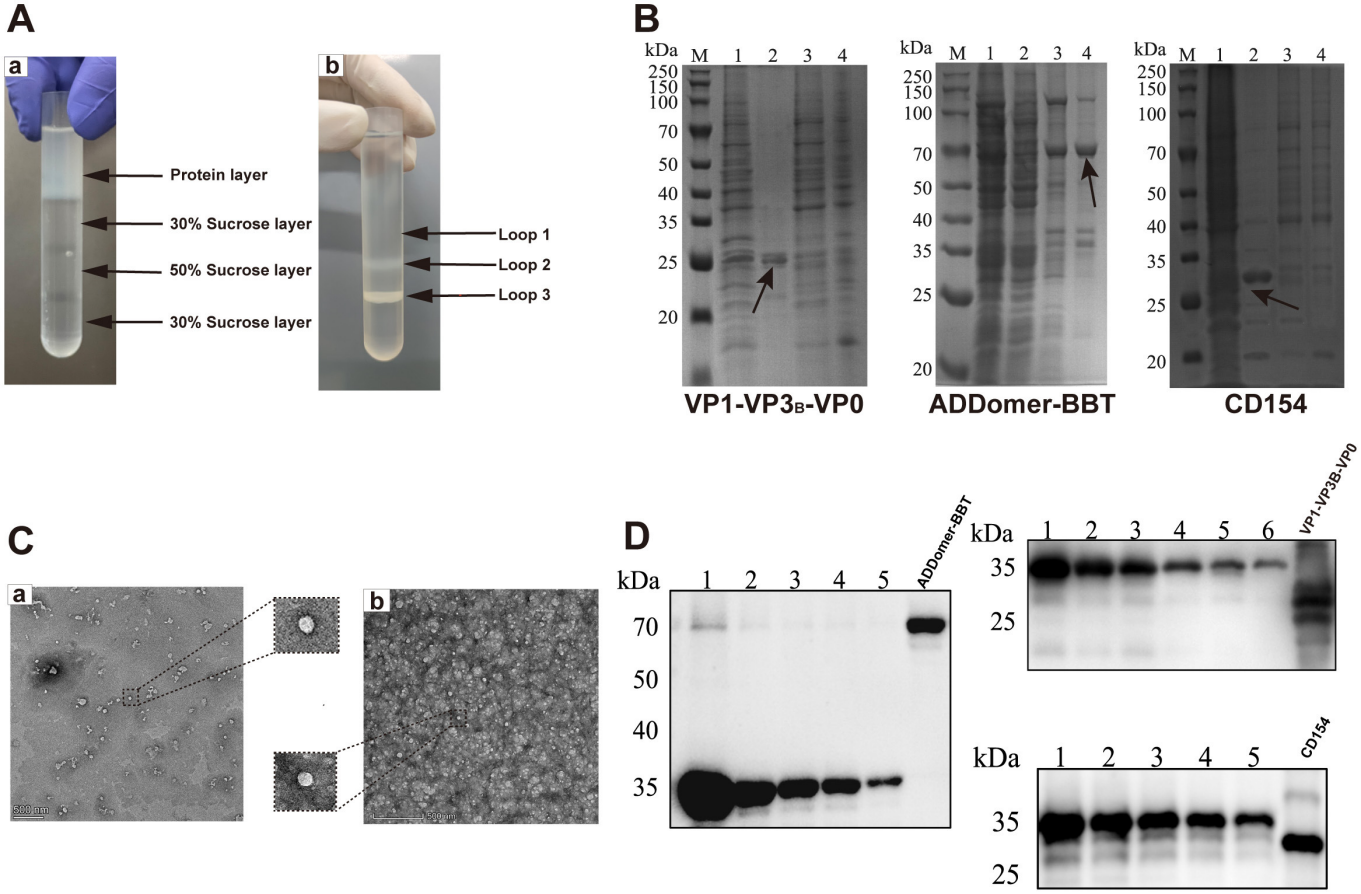
Protein purification, TEM observation, and semi-quantitative analysis. **(A)** Comparison of sucrose gradient purification results. **(B)** Coomassie blue staining before and after purification: M, protein marker; 1, pre-purification; 2, protein sample in loop 1; 3, protein sample in loop 2; 4, protein sample in loop 3. **(C)** TEM images of VLPs: a, VP1-VP3_B_-VP0; b, ADDomer-BBT. **(D)** Semi-quantitative analysis of recombinant proteins, using six standard concentrations (1,000, 500, 250, 125, 62.5, and 31.25 μg/ml His-tag protein).

Purified recombinant proteins obtained under optimal expression conditions were separated by 30%, 50%, and 70% sucrose gradient centrifugation. Protein bands (“protein rings”) were observed in the interlayers of 30%, 30–50%, and 50–70% sucrose (Figure 4A). SDS-PAGE analysis of these layers revealed specific protein bands with minimal non-specific bands: VP1-VP3_B_-VP0 at 24, 25, and 30 kDa; ADDomer-BBT at 71 kD; and CD154 at 30 kDa (Figure 4B). Using 2% uranyl acetate staining, TEM showed that the VLP assembled by VP1-VP3_B_-VP0 was 25–30 nm in diameter, while the VLP assembled by ADDomer-BBT was 30–40 nm (Figure 4C). Recombinant protein concentrations were determined by measuring the gradient of His-tagged standards: VP0, VP1, and VP3_B_ in VP1-VP3_B_-VP0 were 51.01 μg/ml, 117.37 μg/ml, and 83.72 μg/ml, respectively; ADDomer-BBT and CD154 concentrations were 239.04 μg/ml and 371.74 μg/ml (Figure 4D).

### 3.5 Piglet immunization

**Figure 5:**
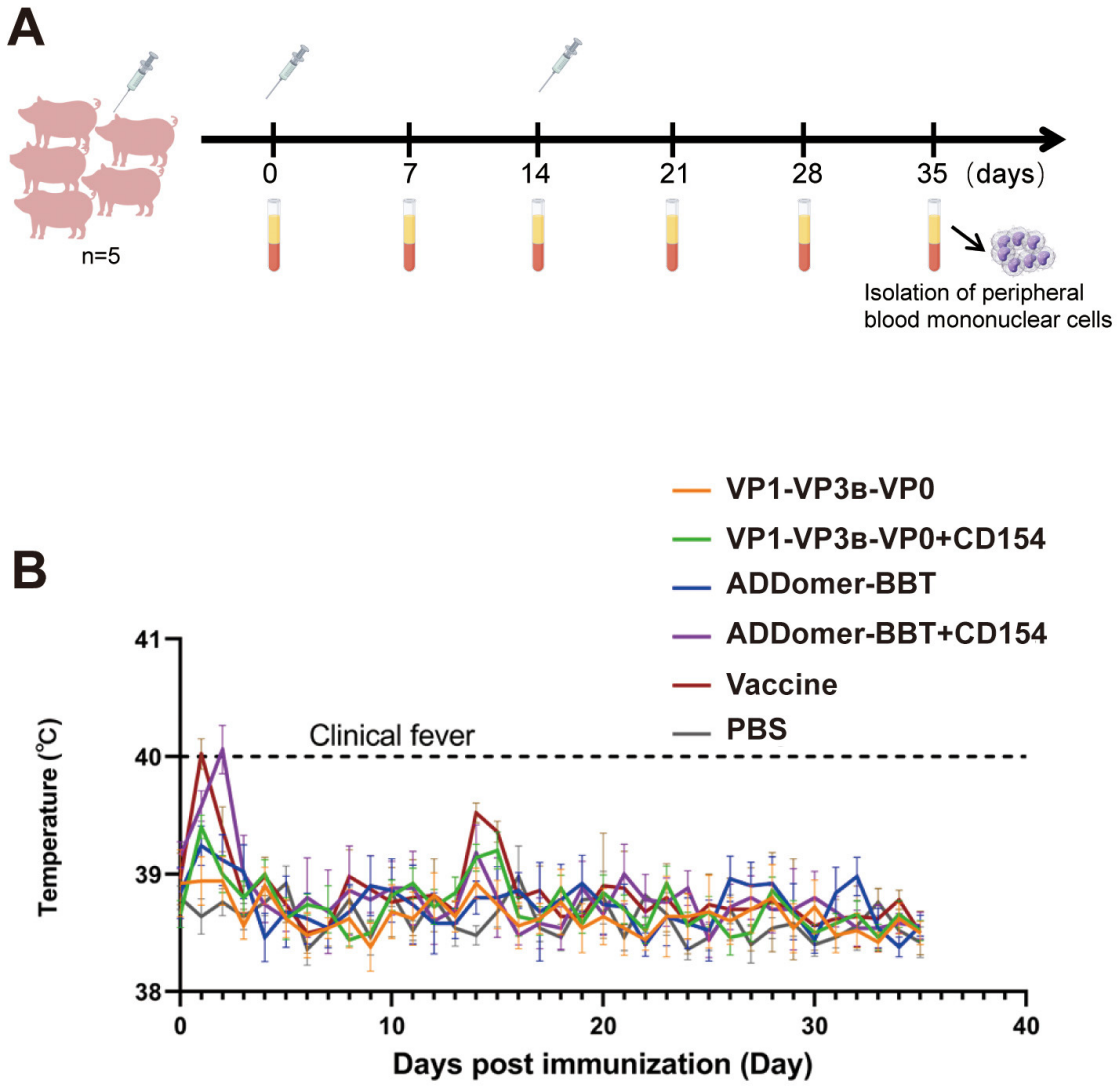
Piglet immunization strategy. **(A)** Piglet immunization plan. **(B)** Rectal temperature changes post-immunization.

The FMDV VLP vaccine formulated with ISA 201 adjuvant formed a white water-in-oil-in-water (W/O/W) emulsion. Upon centrifugation at 3,000 rpm, 4°C for 15 min, no phase separation occurred, indicating the vaccine's stability. Thirty FMDV antigen- and antibody-negative piglets were immunized according to the strategy in Figure 5A. At 35 days post-inoculation, all piglets survived with normal behavior, appetite, and stable body temperature (Figure 5B). No adverse reactions were observed at the injection site.

### 3.6 Detection of FMDV-specific antibodies in piglet serum

**Figure 6:**
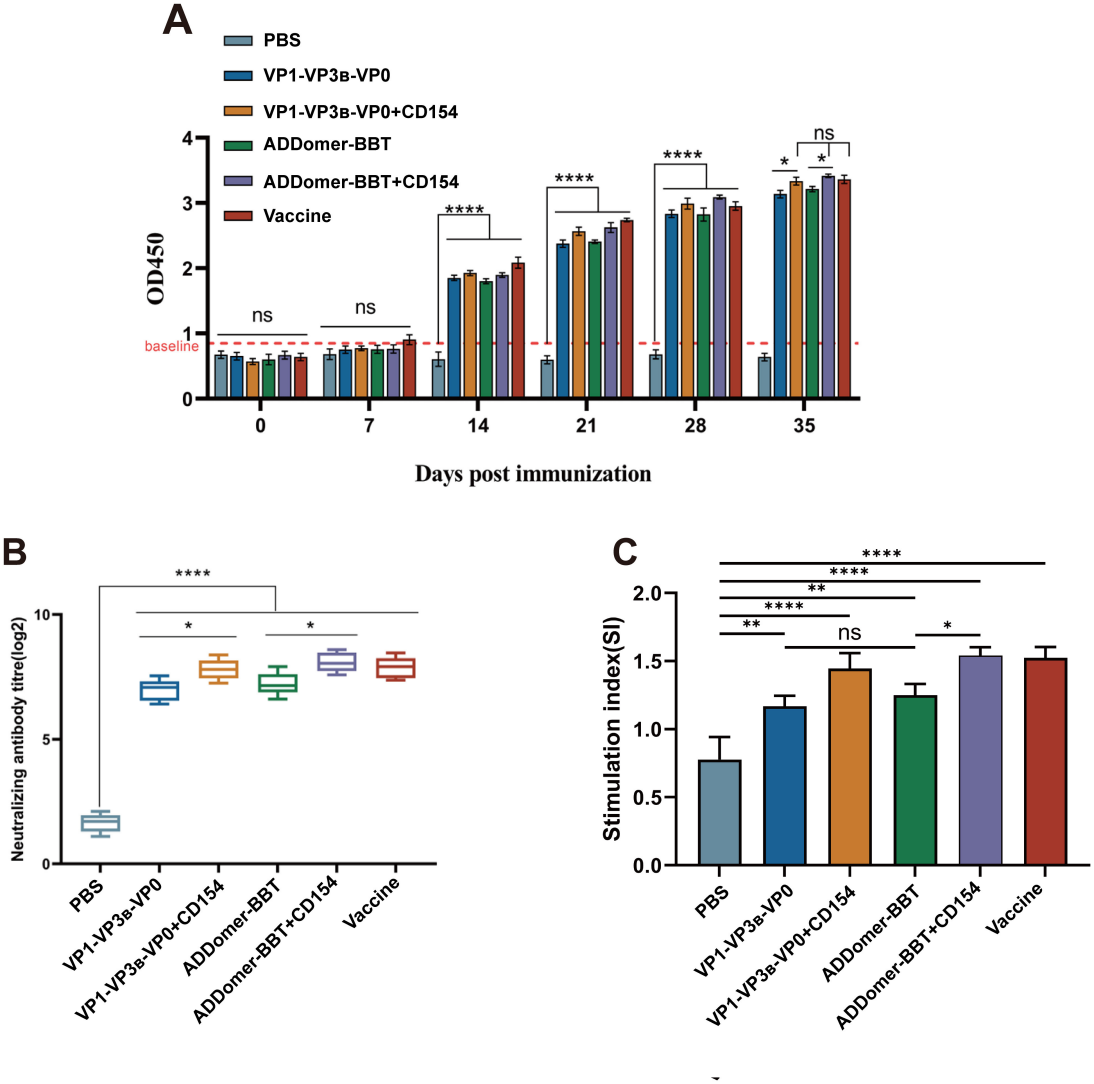
FMDV antibody level detection and peripheral blood lymphocyte proliferation assay. **(A)** Serum FMDV-specific antibody detection in piglets. **(B)** Detection of serum FMDV neutralizing antibodies in piglets. **(C)** Detection of proliferative activity of mononuclear cells in peripheral blood of piglets. ns, *P* > 0.05; *, *P* < 0.05; **, *P* < 0.01; ****, *P* < 0.0001.

Peripheral blood was collected every seven days after the primary immunization to detect serum FMDV-specific antibody levels. At 35 days post-primary immunization, specific antibody levels in the ADDomer-BBT+CD154 vaccine group were higher than the commercial vaccine group, with no significant difference (*P* > 0.05; Figure 6A). The VP1-VP3_B_-VP0 + CD154 group showed antibody levels comparable to the commercial group (*P* > 0.05). Antibody levels in the ADDomer-BBT group were slightly higher than the VP1-VP3_B_-VP0 group, without significant difference (*P* > 0.05). Specific antibody levels were significantly higher in the VP1-VP3_B_-VP0 + CD154 group than in the VP1-VP3_B_-VP0 group (*P* < 0.05), and in the ADDomer-BBT + CD154 group than in the ADDomer-BBT group (*P* < 0.05), indicating that CD154 can enhance the humoral immune response of FMDV VLP-induced immunity in piglets.

### 3.7 Detection of FMDV neutralizing antibodies in piglet serum

To assess protective efficacy, FMDV neutralizing antibody levels in piglet serum were measured 35 days post-primary immunization. As shown in Figure 6B, all vaccine groups showed high neutralizing antibody titers, comparable to the commercial vaccine (*P* > 0.05), except for the PBS group. Neutralizing antibody levels in the VP1-VP3_B_-VP0 + CD154 and ADDomer-BBT + CD154 groups were significantly higher than in the VP1-VP3_B_-VP0 and ADDomer-BBT groups (*P* < 0.05), confirming the ability of CD154 to enhance humoral immune effects.

### 3.8 Proliferative response of peripheral blood lymphocytes in piglets

To evaluate immune response levels post-vaccination, lymphocyte proliferation in piglet peripheral blood was measured using the CCK-8 method. Results indicated that all vaccine groups showed a significantly higher stimulation index (SI) than the PBS group (*P* < 0.01). The ADDomer-BBT group had a slightly higher SI than the VP1-VP3_B_-VP0 group, with no significant difference (*P* > 0.05). VLP vaccine groups containing CD154 showed higher SI than those without CD154 (*P* < 0.05). These findings suggest that vaccines developed in this study significantly enhance the immune response against FMDV in piglets, strengthening their resistance to viral infection (Figure 6C).

### 3.9 Detection of cytokines in piglet serum

**Figure 7:**
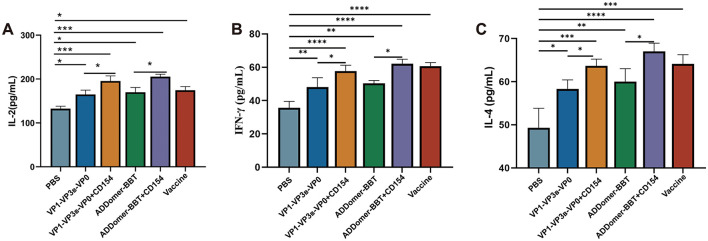
Cytokine secretion levels. **(A)** IL-2 levels in piglet serum. **(B)** IFN-γ levels in piglet serum. **(C)** IL-4 levels in piglet serum. ns, *P* > 0.05; *, *P* < 0.05; **, *P* < 0.01; ***, *P* < 0.001; ****, *P* < 0.0001.

To investigate the host cellular immune mechanisms following vaccination, indirect ELISA kits were used to measure IL-2, IFN-γ, and IL-4 levels in the peripheral blood of piglets at 35 days post-primary immunization. These cytokine levels were assessed to determine the Th1- and Th2-type immune responses induced by the vaccines. The results (Figures 7A, C) indicated that IL-2 and IFN-γ levels in all vaccinated groups were significantly higher than those in the PBS control group (*P* < 0.05). Furthermore, the IL-2 and IFN-γ levels in the VP1-VP3_B_-VP0 + CD154 group were significantly higher than those in the VP1-VP3_B_-VP0 group (*P* < 0.05). Similarly, the ADDomer-BBT + CD154 group exhibited IL-2 and IFN-γ levels that were significantly elevated compared to those in the ADDomer-BBT group. These findings suggest that the VP1-VP3_B_-VP0 and ADDomer-BBT recombinant proteins effectively induce IL-2 and IFN-γ secretion, initiating a Th1-type cellular immune response, while CD154, as an adjuvant, enhances the vaccine's ability to stimulate IL-2 and IFN-γ production.

For IL-4 levels across all vaccinated groups, each group displayed significantly higher IL-4 secretion compared to the PBS control group (*P* < 0.01), and the addition of CD154 further enhanced IL-4 levels (Figure 7B). This result indicates that each VLP vaccine promotes IL-4 secretion, thereby inducing a Th2-type cellular immune response.

## 4 Discussion

FMD is a highly contagious disease that severely affects the health of cloven-hoofed animals. Its control relies primarily on monitoring outbreaks, culling infected animals, and periodic vaccination with FMD vaccines (27, 28). In recent years, traditional inactivated vaccines have played an important role in epidemic control, yet these vaccines have limitations. Thus, the development of genetically engineered vaccines against FMDV is of critical importance for disease eradication.

Numerous studies have demonstrated the promising potential of genetically engineered vaccines using FMDV antigen epitopes. However, researchers have noted that single antigen epitopes are often inefficiently delivered to antigen-presenting cells, susceptible to degradation, and lack pathogen-associated molecular patterns (PAMPs) necessary for effectively activating the immune system; as a result, they frequently require adjuvants (29–31). In light of these challenges, we selected two B-cell epitopes and one T-cell epitope from VP1, as well as FMDV capsid proteins, to prepare FMDV VLP vaccines, with CD154 included as a molecular adjuvant. Currently, various high-yield expression systems are commercially available for producing recombinant VLP vaccines, including mammalian cells, yeast, *E. coli*, and insect cell systems. The insect cell expression system is advantageous over prokaryotic systems (e.g., *E. coli*) due to its superior post-translational modification capabilities, which are crucial for expressing complex recombinant proteins, and it is also more cost-effective than mammalian cell systems, making it ideal for VLP vaccine production (32).

Electron microscopy revealed that both types of VLPs we produced exhibited morphologies resembling natural viral particles. Semi-quantitative analysis of recombinant proteins showed that the expression levels of VP1, VP3_B_, and VP0 were not identical in the co-expressed recombinant VP1-VP3_B_-VP0 protein. Despite the fact that co-expressing multiple proteins can reduce reagent consumption and time costs compared to expressing single proteins, expression levels may vary due to differences in protein physicochemical properties, making it challenging to achieve the desired yield. When specific ratios among target proteins are required in subsequent experiments, further optimization of co-expression conditions is needed, or the proteins should be individually expressed and then mixed in the desired proportions.

Post-vaccination indicators in piglets showed that both VLP vaccines elicited strong cellular and humoral immune responses. IFN-γ, IL-2, and IL-4 are key cytokines that play critical roles in modulating immune responses and are representative markers for evaluating the degree of cellular and humoral immunity induced by a vaccine (33). IFN-γ, mainly secreted by Th1 cells and natural killer cells, activates macrophages, enhances antiviral capacity, promotes antigen presentation, and inhibits viral replication (34, 35). IL-2, primarily secreted by activated T cells, promotes T and B cell proliferation and differentiation, supporting the persistence and efficacy of the immune response, while IL-4, secreted by Th2 cells, stimulates B cell proliferation and antibody production (36–38). Experimental results indicated significant increases in IFN-γ, IL-2, and IL-4 levels in each VLP vaccine group, effectively stimulating both Th1 and Th2 immune responses. Specific antibody detection evaluates whether the vaccine effectively induces pathogen-specific humoral immune responses, while neutralizing antibody testing serves as a key measure of vaccine efficacy by assessing the ability of antibodies to inhibit pathogen infectivity, thus directly reflecting the vaccine's effectiveness in preventing infection and conferring protective immunity. Both VLP vaccines induced high levels of FMDV-specific antibodies and neutralizing antibodies, with neutralizing antibody titers in all groups exceeding the 1:64 threshold, and the ADDomer-BBT+CD154 group showed the highest levels of specific and neutralizing antibodies. As a member of the TNF superfamily, CD154 binds to CD40 receptors, activating B and T cells and enhancing the function of antigen-presenting cells, thereby improving the overall efficiency of immune responses (22, 39). Our study demonstrated that the addition of the CD154 molecular adjuvant enhanced the immunogenicity of the FMDV VLP vaccine, with CD154-adjuvanted groups showing higher levels of specific antibodies, neutralizing antibodies, and cytokines in piglets compared to groups without CD154.

Although animal challenge experiments could not be conducted due to limited resources, our findings indicate that both VLP vaccines show promising protective effects against FMDV, and the addition of CD154 as a molecular adjuvant effectively enhances the immune response, improving the control of FMD. Additionally, the injection dosage also affects the protective effect of vaccines. Some studies have shown that polypeptide vaccine doses (PPVDs) have a dose-dependent protective effect on pigs (40). This implies that selecting an appropriate injection dosage is crucial during vaccine development and application to ensure that the vaccine can effectively induce an immune response and provide adequate protection. Therefore, future research should further explore the immune responses and protective effects at different dosages to provide a more scientific basis for vaccine optimization and application.

## Data Availability

The original contributions presented in the study are included in the article/Supplementary material, further inquiries can be directed to the corresponding author.

## References

[1] El-Shehawy LI Abu-Elnaga HI Rizk SA Abd El-Kreem AS Mohamed AA Fawzy HG. Molecular differentiation and phylogenetic analysis of the Egyptian foot-and-mouth disease virus Sat2. Arch Virol. (2014) 159:437–43. 10.1007/s00705-013-1825-1 24046086

[2] Fukai K Kawaguchi R Nishi T Ikezawa M Yamada M Seeyo KB. Risk of transmission of foot-and-mouth disease by wild animals: infection dynamics in Japanese wild boar following direct inoculation or contact exposure. Vet Res. (2022) 53:86. 10.1186/s13567-022-01106-0 36273214 PMC9587633

[3] Stenfeldt C Pacheco JM Borca MV Rodriguez LL Arzt J. Morphologic and phenotypic characteristics of myocarditis in two pigs infected by foot-and mouth disease virus strains of serotypes O or A. Acta Vet Scand. (2014) 56:42. 10.1186/s13028-014-0042-6 25015718 PMC4105858

[4] Bai XW Li PH Bao HF Liu ZX Li D Lu ZJ. Evolution and molecular epidemiology of foot-and-mouth disease virus in China. Chin Sci Bull. (2011) 56:2191–201. 10.1007/s11434-011-4563-3

[5] Li FT Li Y Ma JR Wu RZ Zou XQ Liu YB. Molecular Evolution, diversity, and adaptation of foot-and-mouth disease virus serotype O in Asia. Front Microbiol. (2023) 14:1147652. 10.3389/fmicb.2023.1147652 36970668 PMC10034406

[6] Ullah A Jamal SM Romey A Gorna K Kakar MA Abbas F. Genetic characterization of serotypes a and Asia-1 foot-and-mouth disease viruses in Balochistan, Pakistan, in 2011. Transbound Emerg Dis. (2017) 64:1569–78. 10.1111/tbed.12548 27484792

[7] Clara Mignaqui A Ruiz V Durocher Y Wigdorovitz A. Advances in novel vaccines for foot and mouth disease: focus on recombinant empty capsids. Crit Rev Biotechnol. (2019) 39:306–20. 10.1080/07388551.2018.1554619 30654663

[8] Wu QH Moraes MP Grubman MJ. Recombinant adenovirus co-expressing capsid proteins of two serotypes of foot-and-mouth disease virus (FMDV): *in vitro* characterization and induction of neutralizing antibodies against. Virus Res. (2003) 93:211–9. 10.1016/S0168-1702(03)00116-3 12782369

[9] Xie Y Li H Qi X Ma Y Yang B Zhang S. Immunogenicity and protective efficacy of a novel foot-and-mouth disease virus empty-capsid-like particle with improved acid stability. Vaccine. (2019) 37:2016–25. 10.1016/j.vaccine.2019.02.032 30808570

[10] Canas-Arranz R de Leon P Forner M Defaus S Bustos MJ Torres E. Immunogenicity of a dendrimer B_2_T Peptide harboring a T-Cell epitope from FMDV non-structural protein 3d. Front Vet Sci. (2020) 7:498. 10.3389/fvets.2020.00498 32851051 PMC7433650

[11] Rangel G Barcena J Moreno N Mata CP Caston JR Alejo A. Chimeric RHDV virus-like particles displaying foot-and-mouth disease virus epitopes elicit neutralizing antibodies and confer partial protection in pigs. Vaccines (2021) 9:470. 10.3390/vaccines9050470 34066934 PMC8148555

[12] Zamorano P Wigdorovitz A Perez-Filgueira M Carrillo C Escribano JM Sadir AM. A 10-Amino-acid linear sequence of Vp1 of foot and mouth disease virus containing B- and T-cell epitopes induces protection in mice. Virology. (1995) 212:614–21. 10.1006/viro.1995.1519 7571431

[13] Mohsen MO Bachmann MF. Virus-like particle vaccinology, from bench to bedside. Cell Mol Immunol. (2022) 19:993–1011. 10.1038/s41423-022-00897-8 35962190 PMC9371956

[14] McFall-Boegeman H Huang X. Mechanisms of cellular and humoral immunity through the lens of Vlp-based vaccines. Expert Rev Vaccines. (2022) 21:453–69. 10.1080/14760584.2022.2029415 35023430 PMC8960355

[15] Zhang L Xu W Ma X Sun X Fan J Wang Y. Virus-like particles as antiviral vaccine: mechanism, design, and application. Biotechnol Bioprocess Eng. (2023) 28:1–16. 10.1007/s12257-022-0107-8 36627930 PMC9817464

[16] Wu P Yin X Liu Q Wu W Chen C. Recombinant T7 phage with FMDV AKT-III strain VP1 protein is a potential FMDV vaccine. Biotechnol Lett. (2021) 43:35–41. 10.1007/s10529-020-03012-x 32989662

[17] Vragniau C Bufton JC Garzoni F Stermann E Rabi F Terrat C. Synthetic self-assembling addomer platform for highly efficient vaccination by genetically encoded multiepitope display. Sci Adv. (2019) 5:eaaw2853. 10.1126/sciadv.aaw2853 31620562 PMC6763337

[18] Chevillard C Amen A Besson S Hannani D Bally I Dettling V. Elicitation of potent Sars-Cov-2 neutralizing antibody responses through immunization with a versatile adenovirus-inspired multimerization platform. Mol Ther. (2022) 30:1913–25. 10.1016/j.ymthe.2022.02.011 35151843 PMC8828441

[19] Cimica V Galarza JM. Adjuvant formulations for virus-like particle (VLP) based vaccines. Clin Immunol. (2017) 183:99–108. 10.1016/j.clim.2017.08.004 28780375 PMC5673579

[20] Matassov D Cupo A Galarza JM A. Novel intranasal virus-like particle (VLP) vaccine designed to protect against the pandemic 1918 influenza a virus (H1N1). Viral Immunol. (2007) 20:441–52. 10.1089/vim.2007.0027 17931114

[21] Qi Y Kang H Zheng X Wang H Gao Y Yang S. Incorporation of membrane-anchored flagellin or *Escherichia coli* heat-labile enterotoxin b subunit enhances the innmunogenicity of rabies virus-like particles in mice and dogs. Front Microbiol. (2015) 6:169. 10.3389/fmicb.2015.00169 25784906 PMC4347500

[22] Diaz A Gonzalez-Alayon I Perez-Torrado V Suarez-Martins M. Cd40-Cd154: a perspective from type 2 immunity. Semin Immunol. (2021) 53:101528. 10.1016/j.smim.2021.101528 34810089

[23] Grewal IS Borrow P Pamer EG Oldstone MB Flavell RA. The Cd40-Cd154 system in anti-infective host defense. Curr Opin Immunol. (1997) 9:491–7. 10.1016/S0952-7915(97)80100-8 9287184

[24] Grewal IS Flavell RA. Cd40 and Cd154 in cell-mediated immunity. Annu Rev Immunol. (1998) 16:111–35. 10.1146/annurev.immunol.16.1.111 9597126

[25] Pamukcu B Lip GYH Snezhitskiy V Shantsila E. The Cd40-Cd40l system in cardiovascular disease. Ann Med. (2011) 43:331–40. 10.3109/07853890.2010.546362 21244217

[26] Wu K Hu W Zhou B Li B Li X Yan Q. Immunogenicity and immunoprotection of Pcv2 virus-like particles incorporating dominant T and B Cell antigenic epitopes paired with Cd154 molecules in piglets and mice. Int J Mol Sci. (2022) 23:14126. 10.3390/ijms232214126 36430608 PMC9694800

[27] Lyons NA Alexander N Staerk KDC Dulu TD Rushton J Fine PEM. Impact of foot-and-mouth disease on mastitis and culling on a large-scale dairy farm in Kenya. Vet Res. (2015) 46:41. 10.1186/s13567-015-0173-4 25889460 PMC4397692

[28] Tewari A Jain B Bhatia AK. Multiplexed diva tests for rapid detection of FMDV infection/circulation in endemic countries. Appl Microbiol Biotechnol. (2020) 104:545–54. 10.1007/s00253-019-10263-w 31832714

[29] Cubillos C de la Torre BG Barcena J Andreu D Sobrino F Blanco E. Inclusion of a specific T cell epitope increases the protection conferred against foot-and-mouth disease virus in pigs by a linear peptide containing an immunodominant B cell site. Virol J. (2012) 9:66. 10.1186/1743-422X-9-66 22416886 PMC3313860

[30] Foged C Hansen J Agger EM. License to kill: formulation requirements for optimal priming of Cd8^+^ CTL responses with particulate vaccine delivery systems. Eur J Pharm Sci. (2012) 45:482–91. 10.1016/j.ejps.2011.08.016 21888971

[31] Soema PC Huber SKR Willems G-J Jacobi R Hendriks M Soethout E. Whole-inactivated influenza virus is a potent adjuvant for influenza peptides containing Cd8^+^ T cell epitopes. Front Immunol. (2018) 9:525. 10.3389/fimmu.2018.00525 29593747 PMC5861146

[32] Hong Q Liu J Wei Y Wei X. Application of baculovirus expression vector system (BEVS) in vaccine development. Vaccines. (2023) 11:1218. 10.3390/vaccines11071218 37515034 PMC10386281

[33] Pinto RA Arredondo SM Bono MR Gaggero AA Díaz PV T. Helper 1/T Helper 2 cytokine imbalance in respiratory syncytial virus infection is associated with increased endogenous plasma cortisol. Pediatrics. (2006) 117:E878–86. 10.1542/peds.2005-2119 16618789

[34] da Silva HB Fonseca R Alvarez JM D'Imperio Lima MR. Ifn-Γ priming effects on the maintenance of effector memory Cd4+ T cells and on phagocyte function: evidences from infectious diseases. J Immunol Res. (2015) 2015:202816. 10.1155/2015/202816 26509177 PMC4609814

[35] Mendoza JL Escalante NK Jude KM Bellon JS Su L Horton TM. Structure of the Ifnγ receptor complex guides design of biased agonists. Nature. (2019) 567:56. 10.1038/s41586-019-0988-7 30814731 PMC6561087

[36] Possamai D Page G Panes R Gagnon E Lapointe R. Cd40l-stimulated B lymphocytes are polarized toward Apc functions after exposure to Il-4 and Il-21. J Immunol. (2021) 207:77–89. 10.4049/jimmunol.2001173 34135061

[37] Rosenberg SA. Il-2: The first effective immunotherapy for human cancer. J Immunol. (2014) 192:5451–8. 10.4049/jimmunol.1490019 24907378 PMC6293462

[38] Wrangle JM Patterson A Johnson CB Neitzke DJ Mehrotra S Denlinger CE. IL-2 and beyond in cancer immunotherapy. J Interferon Cytokine Res. (2018) 38:45–68. 10.1089/jir.2017.0101 29443657 PMC5815463

[39] Mackey MF Barth RJ Jr Noelle RJ. The role of Cd40/Cd154 interactions in the priming, differentiation, and effector function of helper and cytotoxic T cells. J Leukoc Biol. (1998) 63:418–28. 10.1002/jlb.63.4.418 9544571

[40] Jiao J Wu P. A meta-analysis: the efficacy and effectiveness of polypeptide vaccines protect pigs from foot and mouth disease. Sci Rep. (2023) 12:21868. 10.1038/s41598-022-26462-x 36536158 PMC9763257

