# Supplementary material for: Foot-and-mouth disease virus-like particle vaccine incorporating dominant T and B cell epitopes: enhanced immune response in piglets with CD154 molecules
Source: Front Vet Sci. 2025 Feb 19;12:1540102. doi: 10.3389/fvets.2025.1540102 (PMC11879940; doi:10.3389/fvets.2025.1540102)
Supplement: Supplementary file 1 [file Image_1.pdf]

Supplementary Material

1 Supplementary Figures

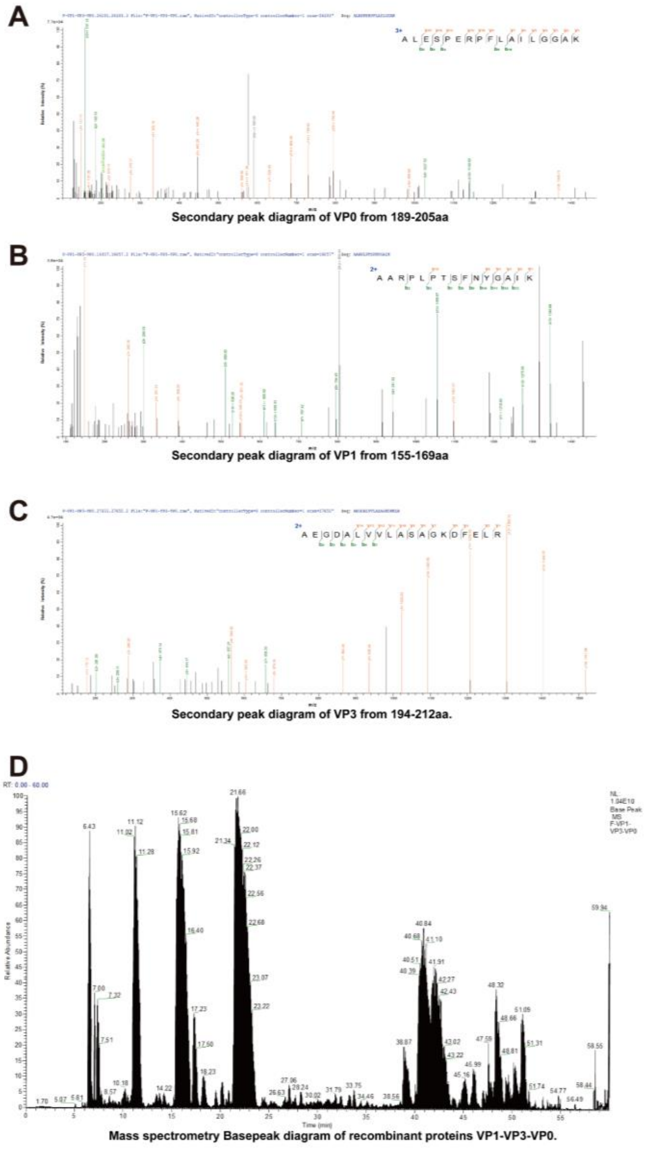

Supplementary Figure 1. (A) The secondary spectrum of the unique peptide segment (189-205 aa) ALESPERPFLAILGGAK from the VP0 protein extracted using the pLabel software, showing that the unique peptide is detectable by mass spectrometry. (B) The secondary spectrum of the unique peptide segment (155-169 aa) AARPLPTSFNYGAIK from the VP1 protein extracted using pLabel software, indicating detection of the unique peptide. (C) The secondary spectrum of the unique

peptide segment (194-212 aa) AEGDALVVLASAGKDFELR from the VP3 protein extracted using pLabel software, showing detection of the unique peptide by mass spectrometry. (D) Basepeak chromatogram of the recombinant VP1-VP3B-VP0 proteins obtained from mass spectrometry.
